# Renal ultrasound provides low utility in evaluating cardiac surgery associated acute kidney injury

**DOI:** 10.1186/s13019-017-0637-x

**Published:** 2017-09-02

**Authors:** Allen Young, Todd Crawford, Alejandro Suarez Pierre, J. Trent Magruder, Charles Fraser, John Conte, Glenn Whitman, Christopher Sciortino

**Affiliations:** 1 Division of Cardiac Surgery, Johns Hopkins University School of Medicine, Sheikh Zayed Tower 1800 Orleans Street, Baltimore, MD 21287 USA; 2 Division of Cardiac Surgery, University of Pittsburgh Medical Center (UPMC) Presbyterian, Suite C-700, 200 Lothrop St. Pittsburgh, Pittsburgh, PA 15213 USA

**Keywords:** Cardiac, Kidney, Renal function failure, Dialysis, Ultrasound, Postoperative care

## Abstract

**Background:**

Renal ultrasonography is part of the algorithm in assessing acute kidney injury (AKI). The purpose of this study was to assess the clinical utility of renal US in postoperative cardiac patients who develop AKI.

**Methods:**

We conducted a retrospective study of 90 postoperative cardiac surgery patients at a single institution from 1/19/2010 to 3/19/2016 who underwent renal US for AKI. We reviewed provider documentation to determine whether renal US changed management. We defined change as: administration of crystalloid or colloid, addition of inotropic or vasopressor, or procedural interventions on the renal system.

**Results:**

Mean age of study patients was 68 ± 13 years. 48/90 patients (53.3%) had pre-existing chronic kidney disease of varying severity. 48 patients (53.3%) had normal renal US with incidental findings and 31 patients (34.4%) had US evidence of medical kidney disease. 10 patients (11.1%) had limited US results due to poor visualization and 1 patient (1.1%) had mild right-sided hydronephrosis. No patients were found to have obstructive uropathy or renal artery stenosis. Clinical management was altered in only 4/90 patients (4.4%), which included 3 patients that received a fluid bolus and 1 patient that received a fluid bolus and inotropes. No vascular or urologic procedures resulted from US findings.

**Conclusion:**

Although renal ultrasound is often utilized in the work-up of AKI, our study shows that renal US provides little benefit in managing postoperative cardiac patients. This diagnostic modality should be scrutinized rather than viewed as a universal measure in the cardiac surgery population.

## Background

Acute kidney injury (AKI) is a common complication in the Intensive Care Unit (ICU) setting and results in a substantial increase in hospital morbidity and mortality [1, 2]. Using the RIFLE criteria, the occurrence of AKI in the ICU setting has ranged from 22% to 36.1% [2, 3]. Renal ultrasound is often utilized in the setting of acute kidney injury to exclude obstructive nephropathy, which occurs in 5–25% of all patients and requires prompt intervention to salvage the kidney [4, 5]. In addition, conventional renal ultrasound can be enhanced by Doppler to assess vasculature patency, renal perfusion, and to rule out renal artery stenosis [6, 7].

In cardiac surgery, the incidence of perioperative renal dysfunction with rising creatinine varies between 1 and 30% with 1% of patients eventually requiring dialysis [8–12]. There is an associated mortality of up to 19% [11, 13, 14]. The renal injury and ischemia occurs due to a multitude of hemodynamic, inflammatory, and nephrotoxic factors involving low cardiac output, hypovolemia, endothelin ET1 mediated vasoconstriction, contact inflammation, and epithelial cell and interstitial edema [12, 15].

Clinical measures to prevent and treat AKI involves avoidance of nephrotoxins and maintenance of normovolemia [12, 15]. In theory, by monitoring renal perfusion through ultrasound, it may allow individualized hemodynamic optimization to limit AKI progression [16]. Management of AKI involves optimizing preload, cardiac output, and organ perfusion with fluids, vasoactive drugs, and inotropes [17, 18]. In the event of anatomical renal injury such as obstruction or stenosis, invasive surgery and revascularization may be necessary to prevent further renal injury.

Acute kidney injury has a high morbidity and mortality leading to lengthened hospital stays and financial costs [19]. However, standardized preventative and treatment strategies in the ICU setting for AKI are still strongly debated. Thus, the goal of this study is to show how frequently renal ultrasound is able to detect obstruction or decreased perfusion in the postoperative cardiac population and thereby modify clinical management.

## Methods

We conducted a retrospective review of the electronic medical records and renal sonographic results for patients admitted to the Intensive Care Units at Johns Hopkins Hospital between 1/19/2010 to 3/19/2016. Johns Hopkins Medicine Institutional Review Board approved this study and granted a waiver of authorization.

Inclusion criteria included all adult patients (>18 years old) who underwent open-heart surgery (AV Replacement, AV replacement and CABG, AV replacement and MV replacement, CABG, MV Repair, MR Repair and CABG, MV Replacement and CABG, and MV Replacement) and later developed AKI that was evaluated by bedside renal ultrasound.

Acute kidney injury was defined according the RIFLE criteria with a 2-3× increase in serum creatinine and/or urine output <0.5 mL/kg per hour for 12 h.

Pre-existing Chronic Kidney Disease (CKD) was further classified based on the scale developed by the Kidney Disease Outcomes Quality Initiative (KDOQI) according to the patients’ pre-operative Glomerular Filtration Rates (GFR).

The renal ultrasound examinations were performed by licensed ultrasonographers at the bedside and reviewed by board certified radiologists.

A number of variables were recorded including the patient age, sex, underlying kidney disease, indication for ultrasound, renal function test results, sonographic findings, and clinical management.

Change in clinical management was chronicled if patients received crystalloid or colloid fluids, addition of inotropic or vasopressor medications, or procedural interventions on the renal vasculature or collecting system.

## Results

A total of 90 patients (men, 64.4%) were included in this study. The patient age ranged from 21 to 88 with the mean age of 68 ± 13 years. 48 patients (53.3%) had a preoperative diagnosis of underlying kidney disease with 23 of those patients having stage IIIA CKD, 19 patients with stage IIIB CKD, and 6 patients with stage IV CKD.

A summary of demographic patient data is listed in Table 1.

**Table 1:** Demographics

Demographic	Number of studies	% of Total (*n* = 90)
Gender	58 Men	64.4 Men
Kidney disease	48	53.5
GFR 60–90+	42	46.7
CKD IIIA (GFR 45–59)	23	25.6
CKD IIIB (GFR 30–44)	19	21.1
CKD IV (GFR 15–29)	6	6.7
AV Replacement Only	20	22.2
AV Replacement +CABG	9	10
AV and MV	2	2.2
MV Replacement Only	8	8.8
MV Repair Only	3	3.3
MV Repair +CABG	5	5.5
MV Replacement +CABG	2	2.2
CABG Only	41	45.5

Comparing preoperative creatinine to maximum creatinine during ICU hospital stay, the rise in creatinine ranged from 1 to 7× increase with mean increase of 2.43× ± 1.19. The indication for all 90 renal ultrasounds was acute renal injury consisting of elevated creatinine and/or oliguria following cardiac surgery.

A summary of pathologic and incidental sonographic findings is listed in Table 2.

**Table 2:** Renal ultrasound findings

Renal sonographic findings	Number of studies	% of Total (*n* = 90)
Benign	12	13.3
Simple cysts	31	34.4
Non-obstructing calculi	3	3.3
Vascular calcifications	2	2.2
Increased echogenicity/cortical thinning (medical renal disease)	31	34.4
Hydronephrosis	1	1.1
Poor visualization	10	11.1
Obstructing calculi	0	0
Renal artery stenosis	0	0

Forty-eight patients had benign or incidental findings (simple cysts, non-obstructing calculi, vascular calcifications) on ultrasound.The most common abnormal sonographic findings was parenchymal or medical renal disease (31/90; 34.4%) as indicated by increased echogenicity or cortical thinning. Mild hydronephrosis was found in 1 patient without CKD (1.1%) who exhibited grade 1 right-sided hydronephrosis. 10 patients had poor visualization on their ultrasound due to body habitus and poor cooperation. No patients were diagnosed with renal artery stenosis or obstructive calculi on ultrasound.

Clinical decisions were made by ICU attendings based on creatinine trends, volume status, and urinary output. Initial supportive management of the postoperative patients was unchanged throughout the ICU stay for 86 (95.5%) of the patients, which involved diuretics (30 patients; 31.1) and auto-diuresis (56 patients; 35.6%). Renal consultation was obtained in 40 (44.4%) of patients. Clinical management was altered in only 4 patients (4.4%), which included 3 patients (1 without CKD, 2 with CKD stage IIIA) that received a fluid bolus and 1 patient with CKD stage IIIB that received a fluid bolus and initiation of inotropic support. No vascular or urologic procedures resulted from the US findings.

## Discussion

Although acute kidney injury is very common in severely ill hospitalized patients and renal ultrasound is effective in ruling out hydronephrosis, urinary tract obstruction, and poor perfusion, their universal use in all patients may cause unnecessary financial costs and subject patients to unneeded examinations.

Our institution’s data clearly demonstrates a low incidence of obstructive or stenotic renal injury and that obtaining universal renal sonography for all postoperative cardiac patients in the setting of AKI did not significantly alter clinical decision-making. However, for those patients who did have altered clinical management, there is a preponderance of patients with chronic kidney disease suggesting that renal sonography can beneficial for those with pre-existing kidney disease.

In a similar study done by Keyserling et al. [20] 105 ICU patients with acute kidney injury (demonstrated by serum creatinine level of 1.5 mg/dL or greater or elevation of serum creatinine level to 20% of baseline or higher) were reviewed. Of the 105 renal sonograms, only 1 showed evidence of mild hydronephrosis, which was not explored further. Their findings were consistent with our data, which showed majority (32/104; 30.5%) having medical renal disease and the remaining showing benign, incidental findings that did not modify clinical management.

In another study by Brivet et al., [21] only 4% (16/360) of patients in an intensive care unit with acute renal failure had a post-renal or obstructive cause, with most cases (282/360; 79%) being secondary to intrinsic renal causes.

Finally, Podoll et al. [22] studied 800 patients over a 3-year period for AKI and hydronephrosis was detected in slightly more than 5% with only 2.3% due to urinary tract obstruction. Moreover, the majority of hydronephrosis cases were mild and considered incidental. However, their study did show that severe characteristics such as age greater than 65, prior abdominal malignancy, prior pelvic or renal surgery, PID, abdominal trauma were all statistically significant in increased hydronephrosis, which is consistent with previous reports from Licurese et al. [23] stating that abdominal abnormality and neoplasm confer a higher risk of detecting hydronephrosis on ultrasound.

Together, our data suggest that renal ultrasound may not be indicated for every patient who presents with AKI and should be reserved for those with predisposing factors for obstructive uropathy and renal artery stenosis. At-risk patients include those with a history of intra-abdominal/pelvic malignancy, palpable abdominal or pelvic mass, pregnancy, past history of nephrolithiasis, bladder outlet obstruction, recent pelvic surgery/trauma, suspected renal sepsis, atherosclerosis, or fibromuscular dysplasia [24, 25].

Future studies may be needed to incorporate Renal Resistive Index (RRI) with conventional ultrasound results to improve monitoring and clinical management of at-risk postoperative patients. RRI measures renal artery resistance to blood flow and can be a useful marker of kidney injury and severity [26]. Studies have shown that RRI >0.70 was significantly associated with increased acute renal failure and mortality in the cardiothoracic population (*p* = 0.008) [26, 27]. Thus, RRI with conventional renal ultrasound may be useful to predict and manage acute renal failure [28, 29].

Limitations for this study involve a retrospective study, non-standardized clinical management decision making based on ICU attending preference, the lack of renal resistive indices reported on kidney renal ultrasounds at our institution, sonographic exams obtained by ultrasonographers who may have differing levels of technical skill, and occasional poor visualization due to body habitus and poor patient cooperation.

## Conclusion

Although renal ultrasound is a common diagnostic tool used to evaluate and rule out renal complications in the ICU, there is little evidence to suggest their beneficial impact on altering clinical decision-making. Thus, for management of acute kidney injury in postoperative cardiac patients, ICU providers should utilize renal ultrasound based on reviewing predisposing factors, including pre-existing kidney disease, rather than as a universal measure.

## Abbreviations

AKI: Acute kidney injury
AV: Aortic Valve
CABG: Coronary Artery Bypass-Grafting
ICU: Intensive Care Unit
MV: Mitral Valve
RIFLE: Risk, Injury, Failure Loss of kidney function, and End stage kidney disease
RRI: Renal Resistive Index
US: Ultrasond
